# Perceptions of virtual clinical learning in dentistry: Understanding student views on virtual dental clinics

**DOI:** 10.1186/s12909-025-07124-8

**Published:** 2025-04-21

**Authors:** Kiran Rehman, Omer Sheriff Sultan, Muneer Gohar Babar, Fareeza Marican, Syed Sarosh Mahdi

**Affiliations:** 1 Division of Restorative Dentistry, IMU University, Kuala Lumpur, Malaysia; 2 Division of Restorative Dentistry, Missouri School of Dentistry and Oral Health, A.T. Still University, Kirksville, USA; 3 Division of Clinical Oral Health Sciences, IMU University, Kuala Lumpur, Malaysia; 4 INCEIF University, Kuala Lumpur, Malaysia

**Keywords:** Dental Education, Virtual Dental Education, Clinical Simulation, E-Learning in Dentistry, Virtual Dental Clinic

## Abstract

**Background:**

The Virtual Dental Clinics (VDCs) are designed in an interactive mode for undergraduate dental students to expose them to dental clinical cases in the form of didactic learning rather than experiential learning. VDCs simulate realistic dental practice scenarios within a virtual environment, providing students with opportunities to engage in clinical decision-making, patient interactions, and procedural simulations.

**Aim:**

This study aims to assess students’ perceptions regarding the usefulness of Virtual Dental Clinics (VDCs) in simulated clinical training using a validated questionnaire. The study seeks to assess student perceptions on usefulness, satisfaction, ease of learning and ease of use of Virtual Dental Clinics as a clinical teaching and learning tool.

**Methodology:**

The Virtual Dental Clinics were designed on themes from sub-specialities of dentistry. Themes for clinical case scenarios were selected and designed with the guidance of specialist faculty and the E-Learn department of International Medical University. “A total of 29 Year 3 dental students participated in the study after using the VDC for two weeks. Year 3 dental students were included, as they recently began clinical postings, lacking exposure to advanced clinical situations. A pre-piloted and validated questionnaire named the ‘USE questionnaire’ was utilized to assess student perceptions regarding the usefulness, ease of use, ease of learning, and satisfaction with VDCs.”

**Results:**

Data analysis showed that students expressed significantly different opinions regarding the domains Usefulness & Ease of Learning (mean difference 0.48, *p* < 0.001) as well as Usefulness and Satisfaction (mean difference 0.43, *p* < 0.001). The significant results for the usefulness domains suggest that although the tool is effective in helping the user achieve their task, there is still room for improvement in making it more user-friendly and easier to learn as well as in overall user satisfaction. Our study evaluated the impact of Virtual Dental Clinics (VDCs) on student perceptions. Data analysis using Wilcoxon signed rank test with Bonferroni correction and found significant differences between ‘Uselessness’ and other domains, which demonstrated the perceived utility of VDCs as an educational tool in the clinical setting. Importantly, uselessness was rated significantly higher compared to ‘Ease of use (*p* < 0.001), ‘Ease of learning’ *p* < 0.001) and satisfaction (*p* < 0.001). No significant differences were observed between ‘Ease of use, ease of learning and satisfaction’ (*p* > 0.05). The results demonstrate VDCs efficacy in supporting educational needs of students. These findings suggest that while users perceived the tool as effective in task completion (usefulness), further improvements may be required to improve its ease of use, learnability, and overall satisfaction. The lack of significant differences among the latter three domains may indicate a comparable user experience in those aspects.

**Conclusion:**

This study has provided an understanding of student perception across the various aspects of usefulness, satisfaction, ease of use, and ease of learning in virtual dental clinics. The significant differences between some domains highlight variability in end-user experience, providing rationale for future improvements and optimization of end-user experience.

## Introduction

Transformations are essential in the realm of education. Conventional teaching methods, at times, fail to align with the individual learning style of each learner. The main aim of curricular design in dentistry is to successfully train proficient and safe dental practitioners [1]. In dentistry, the students undergo rigorous preclinical training before advancing to the clinical aspect [2]. Pre-clinical students predominantly study basic sciences as didactic teaching and practical work is done on models and phantom heads in the simulation laboratory. During the clinical training phase, students are exposed to patients through hands-on training under direct and indirect supervision from teaching faculty in the various dental disciplines. However, transitioning from pre-clinical to clinical dentistry can be stressful for learners as they have not yet been exposed to real-life patients [2]. Dental students have identified clinical training as the most stressful part of their dental curriculum and clinical intensive years of the dentistry courses as the most stressful period of the whole graduate program [3–5].

Developing excellent clinical abilities also requires the clinicians’ capacity to objectively self-examine outcomes, identify strengths, weakness and areas for improvement, and implement strategies to accomplish these goals [3]. Identifying fundamental clinical mistakes comes with developing a student’s self-reflection capabilities, allowing for better outcomes. The effectiveness of various methods in clinical training are critical in determining the competence and confidence of future dental practitioners [6, 7]. Simulation based education for dental training has been widely used for a long time with promising results in equipping the students with adequate pre-clinical and psychomotor skills. Issenberg et al. reported in their review that simulation-based education is effective in medical education for clinical care settings [8]. Last few decades have witnessed many advancements in the creation and delivery of dental and medical curricula [9, 10]; one such innovation gaining momentum is the incorporation of virtual dental clinics (VDCs), which offers a novel approach to supplement traditional clinical training methods [2].

The advantages of digital learning are mainly related to its accessibility, enhanced quality of images, ease of use and time management [11]. There are a wide range of digital products now available in the market for dental education which range from e-Learning platforms, simulation software, mobile applications to state-of-the art virtual-reality simulators which offer a near real-time experience of what it is like to be conducting dental procedures on a real patient [12]. The idea of virtual dental clinics (VDCs) comes from the wider concept of digital dentistry, which combines various digital technologies to enhance pre-clinical learning experience for students and teachers. The basic aim is to introduce dental students to clinical scenarios and situations through didactic means instead of experiential learning, using virtual dental clinics. VDCs allows clinical instructors to calibrate clinical cases from simple to complex according to the training needs of the students. VDCs are therefore an effort to bridge the gap between theoretical pre-clinical training and practical application of these skills in the clinical setting.

The VDCs are based on the constructivist principles [13, 14] and provide an interactive environment where the students can immerse themselves with simulated clinical cases and situations [15]. Students can work on complex cases, make clinical decisions and evaluate the outcomes of those decisions; all in a safe virtual environment [16, 17].

Therefore, the VDCs have multiple benefits. The students can fine tune their diagnostic skills and upgrade their knowledge in a controlled, safe and simulated learning environment, which mimics real clinical setting of dental chairside. This gives them the confidence to face challenges in clinical situations where they have direct patient interactions and difficult case situations to diagnose. VDCs also have the additional benefit of providing instant feedback on the performance of the students, which consequently allow learners to specifically target areas that require improvement and more intensive practice. By the time the students enter clinical dentistry, they are expected to be trained to a level where they can create appropriate treatment plans and self-evaluate. These are critical steps to help them practice safe clinical dentistry, by the end of their 5-year undergraduate course.

While the potential benefits of VDCs in dental education are evident, it is critical to carefully assess their usefulness and impact on student learning outcomes [18]. Questionnaire-based evaluation provides a robust methodology for gathering feedback from students regarding their experiences with VDCs, illustrating strengths and weaknesses and highlighting areas for further improvement [19]. Educators can improve the instructional value and efficacy of VDCs by analysing the data collected through the questionnaire. The USE (Usefulness, Satisfaction, and Ease of Use) questionnaire developed by Arnold M. Lund [20, 21] has a broad scope of application. It is non-proprietary and, therefore, available online, free of charge.

This study aims to assess students’ perceptions regarding the usefulness of Virtual Dental Clinics (VDCs) in simulated clinical training using a validated questionnaire. The study seeks to assess student perceptions on usefulness, satisfaction, ease of learning and ease of use of Virtual Dental Clinics as a clinical teaching and learning tool.

Secondary aim.

- To assess the VDCs as an enhancement to simulated learning, by providing a more holistic approach to case-based learning.

## Materials and methods

This study was designed in two phases, with a team of dental specialists and faculty members as well as members from the E-learn department at IMU university. The Virtual Dental Clinics were designed on themes from sub-specialities of dentistry which included themes of Oral Medicine and radiology, Oral pathology, Oral and maxillofacial surgery and endodontics.

### Phase 1

#### Development of virtual dental clinics

The Virtual Dental Clinics were created by collective effort and collaboration of subject experts from various domains of dentistry with the guidance of eLearning department at IMU University. During the developmental phase, subject experts from disciplines of endodontics, periodontology, oral radiology, oral pathology and oral and maxillofacial surgery developed the clinical case scenarios and storylines for the virtual dental clinics. The cases were selected according to the students’ levels of understanding and the most observed clinical situations at the Oral health centre at IMU university.

The themes of clinical dentistry included in the study were carefully chosen as those which are not commonly seen in clinical practice for year 3 students.

The cases that were developed included:

Endodontics: A case of an Endo Perio lesions.

Oral and Maxillofacial Surgery: Wisdom tooth extraction.

Oral Medicine and radiology: Swelling in the floor of the mouth.

Oral Pathology: A case of swelling of jaws (Dentigerous cyst).

Periodontology: A case of periodontal abscess.

The storylines were then shared with team members from the E-learn department, who together with subject experts converted them to videos, simulations and animations, which ensured an immersive learning experience for the students. The entire process including the review and revision with experts was completed in 3 months. The student engagement was facilitated due to the immersive nature of the whole exercise, the engagement spanned for multiple sessions and students were required to complete designated clinical scenarios and quizzes.

We chose the USE questionnaire due to its wide use in previous studies and validation. The USE questionnaire details comprehensive view of user experience by encompassing all critical aspects like ease of learning, usefulness, and satisfaction. Given its durability, universal use and validation [22], the USE questionnaire was deemed as the best choice to base the study on by the authors. The questionnaire uses a Likert scale consisting of 30 Questions. The questions are rated on a seven-point Likert scale ranging from 1 to 7, where 1 being “strongly disagree” to 7 rated as “strongly agree”. N/A (Not applicable is also kept as an option.) The questionnaire is divided into 4 domains that are: ease of use, ease of learning, usefulness and satisfaction. The respondents were also allowed to comment on each domain. Respondents were also requested to identify 3 strengths and 3 weaknesses at the end of the questionnaire.

A systematic approach was required for the successful development of and implementation of Virtual Dental Clincs (VDCs) which follows several steps starting with identification of learning outcomes, analysis of the content, design formation and evaluation of its utility as a teaching aid. The team developed interactive virtual dental cases tailored on the ADDIE model (Analysis, design, development, implementation and evaluation) by integrating relevant instructional design framework as well as utilizing gamified learning theory [23].

**Fig. 1:**
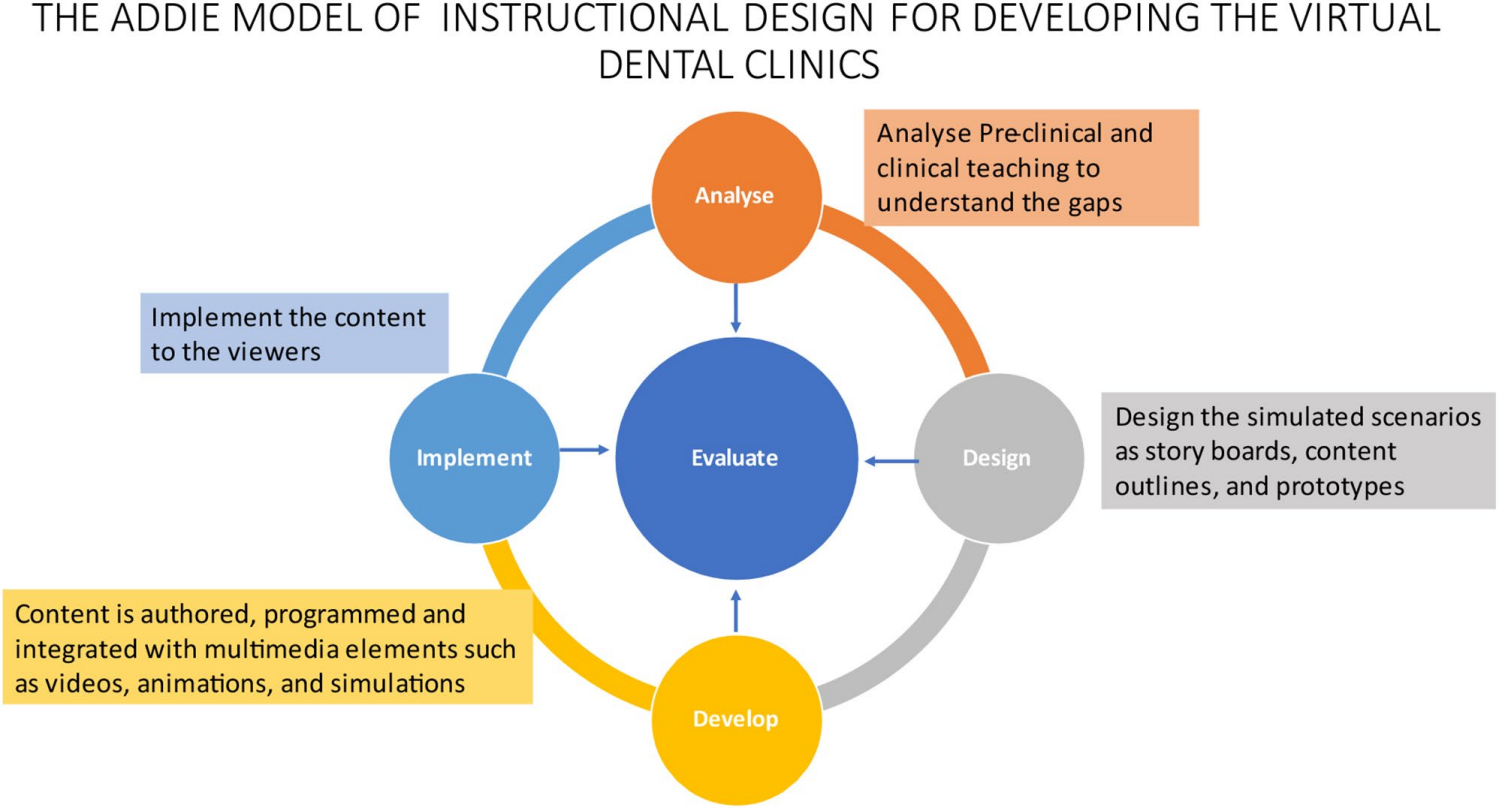
Flowchart representing the phases of development for the virtual Dental Clinics using the ADDIE model

The ADDIE model (Fig. 1) has been used previously as the framework for developing content as it offers a structed and systematic approach to the development of VDCs. The model consists of five stages: Evaluation, implementation, development, design & analysis. Educational systems require pedagogical frameworks to achieve successful implementation and development of complex skills sets required in this competitive environment [15].

The ADDIE model ensures that the interactive content is developed consistently and effectively, promoting learner engagement and improving the learning experience. The interactive VDC was developed in 3 months, including review and evaluation sessions with the subject matter expert to ensure the final product is high quality and meets the learning objectives.

**Fig. 2:**
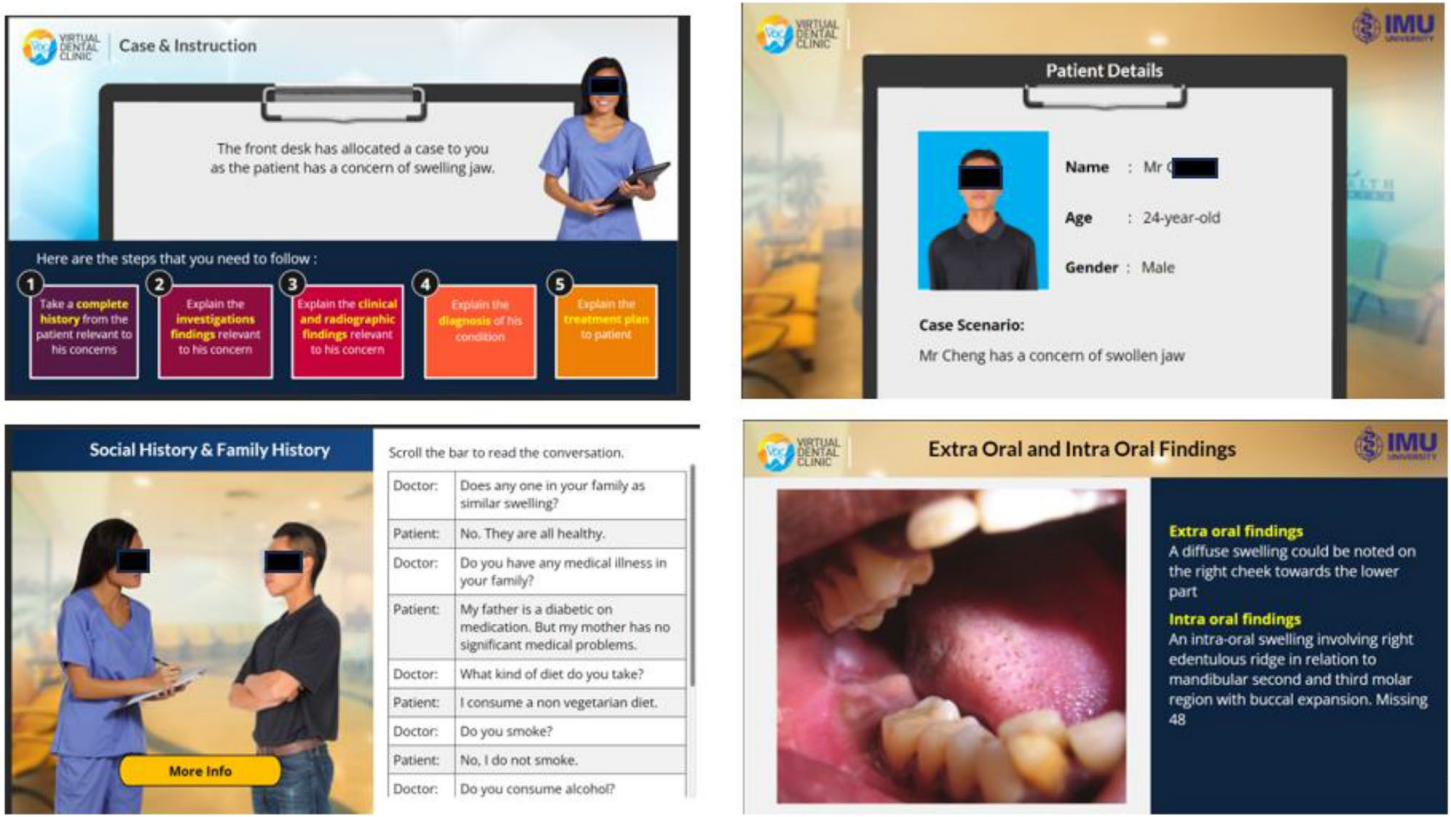
Images showing the various stages of the Virtual dental clinic in an interactive mode

All images were generated on Articulate 360, for which the institution has a paid subscription and licence to use. Figure 2 shows a sample of the design of the VDCs which was developed as a collaborative effort by subject matter experts from school of dentistry and e-learn graphic design team experts. The faces shown in Fig. 2 are not real humans but animations. The development of each VDC was regularly reviewed and evaluated to ensure authenticity and integrity of the content. The completed course was published in SCORM format and later uploaded to the Learning Management System (LMS) for the students to access.

## Phase 2

### Ethical approval

This study was reviewed and approved by the IMU Joint-Committee on Research & Ethics, International Medical University (Ethics Committee/IRB Reference Number: 4.3/JCM-274/2023), as part of the Bachelor of Dental Surgery program.

### Inclusion and exclusion criteria

The students from Year 3 who had just started clinical dentistry were required to undertake the Virtual Dental Clinics. These students have only a few months of experience in treating real-life patients. The only other form of training and teaching these students were delivered on history taking and examination, was through lecture based didactic environment, and some self-directed learning, on their own peers as simulated patient scenarios.

Any student with exposure of more than 3 months in clinical dentistry was excluded from the study.

### Sample size calculation and exposing the students to the virtual dental clinics

Yamane’s formula: n = N/(1 + N(e)2 was used to calculate the appropriate sample size. The total number of students was determined to be 76 according to the formula, but the study was able to recruit only 29 students for the study. Students from Year 3 were included in this study, as they have just started their clinical posting and have not yet been exposed to a variety of advanced clinical situations. The students who agreed to be part of the study were exposed to the Virtual dental clinics for a period of 2 weeks within which they had to complete all the provided Virtual Dental Clinics. The VDCs required students to take history, conduct investigations, reach a diagnosis and formulate a treatment plan. Thefinal sample was smaller than the one calculated by the Yamane formula due to low response rate from the students, but despite the smaller cohort, the study was conducted to provide valuable information on the novel technology and its current acceptance by the students. The sample size might have been small, but it maintains sufficient power for measuring meaningful impact within the context of this research. The insights gained from this study warrant further investigation.

**Fig. 3:**
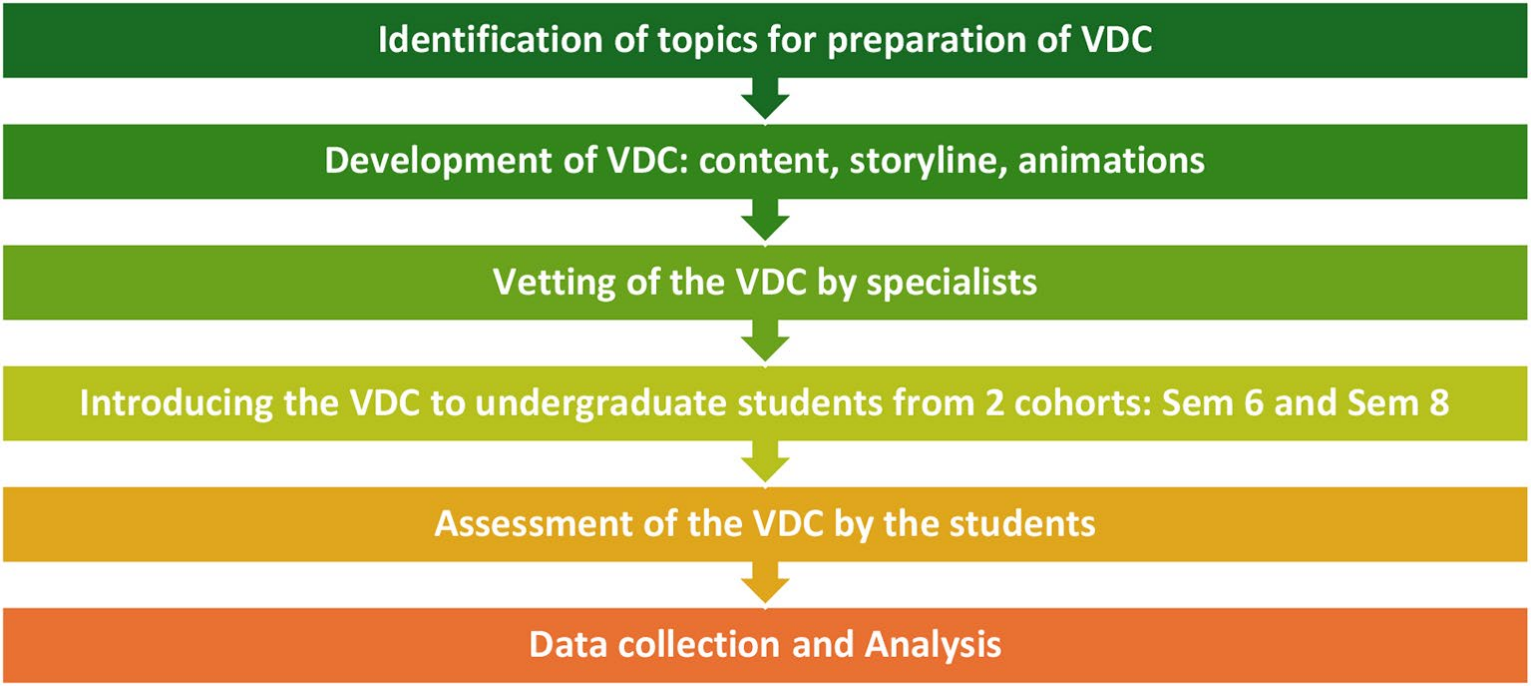
Summary of Steps Involved

The VDCs were provided to them on their E-Learning platform. The students could attempt the VDCs multiple times within the 2-week time frame. The VDCs were viewed, and quizzes were completed as part of the interactive videos. Once completed at the end of the 2-week period, the students were asked to provide their feedback after the completion of the VDC, for which a previously used and piloted questionnaire was utilized to ascertain the usefulness of virtual dental clinics in improving student outcomes regarding the clinical cases presented. This “USE questionnaire” is a validated and widely used tool for data collection. The USE questionnaire contains a total of 30 questions, which include five domains: usefulness, ease of use, ease of learning, and satisfaction. A Likert scale ranging from 1 (Strongly Disagree) to 7 (Strongly Agree) was used to rate each question within these domains [21]. Figure 3 represents a summary of all the steps involved from identification of topics to final data collection and analysis involved in this study.

### Data analysis

Given the small sample simple (*n* = 29), there was possibility of deviations from normality in the data distribution, that is why a non-parametric approach was adopted after suggestions from external reviewers. The Wilcoxon-signed-rank test, which is a non-parametric alternative to the paired t-test was utilized to investigate the differences between paired domains. The test doesn’t require the data to be normally distributed and is suitable for analysing ordinal, interval and dependent data in small sized samples. Finally, *p*-values were calculated for each domain comparison to examine the statistical significance of the observed differences between the means of various domains. Statistical significance in knowledge and other variables was set at < 0.05.

## Results

**Fig. 4:**
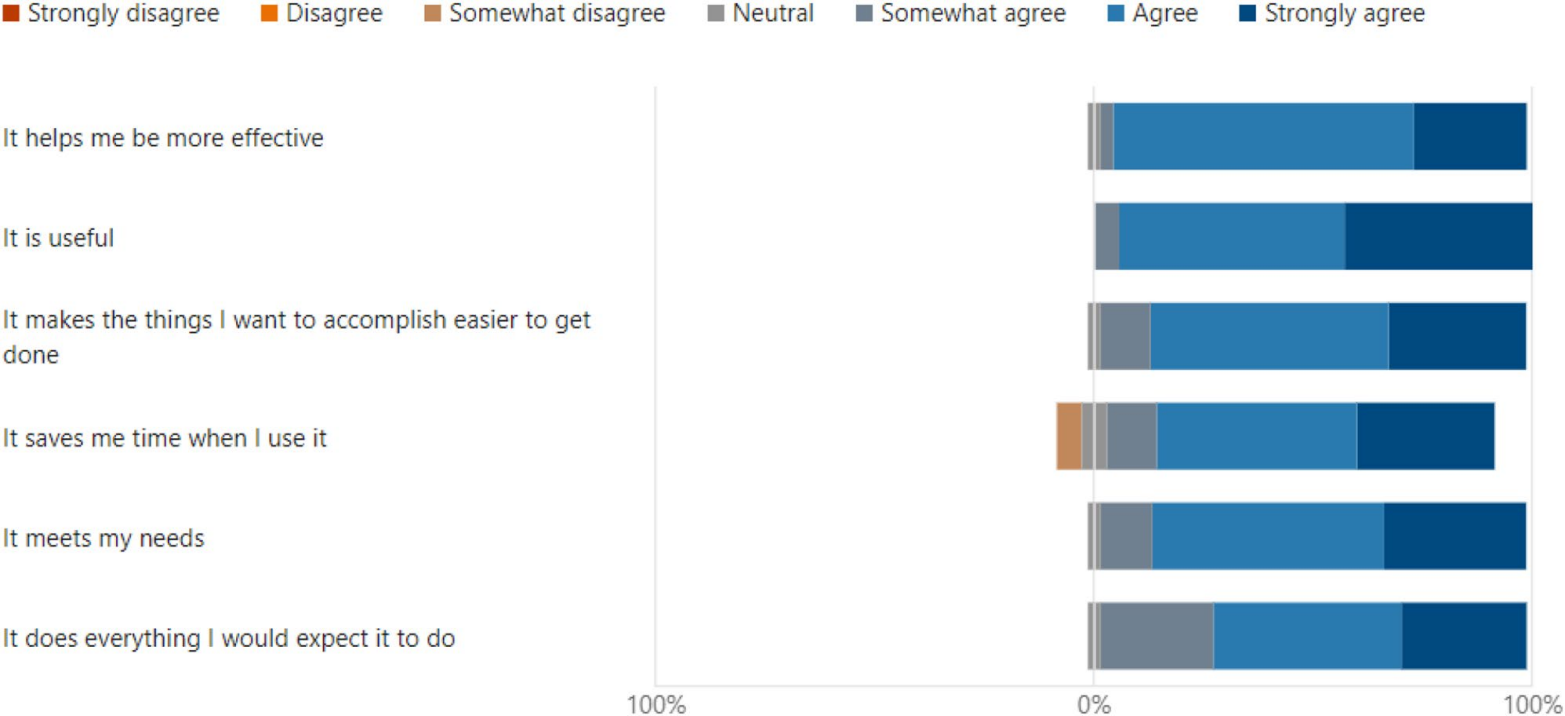
Results for “Usefulness” domain

**Fig. 5:**
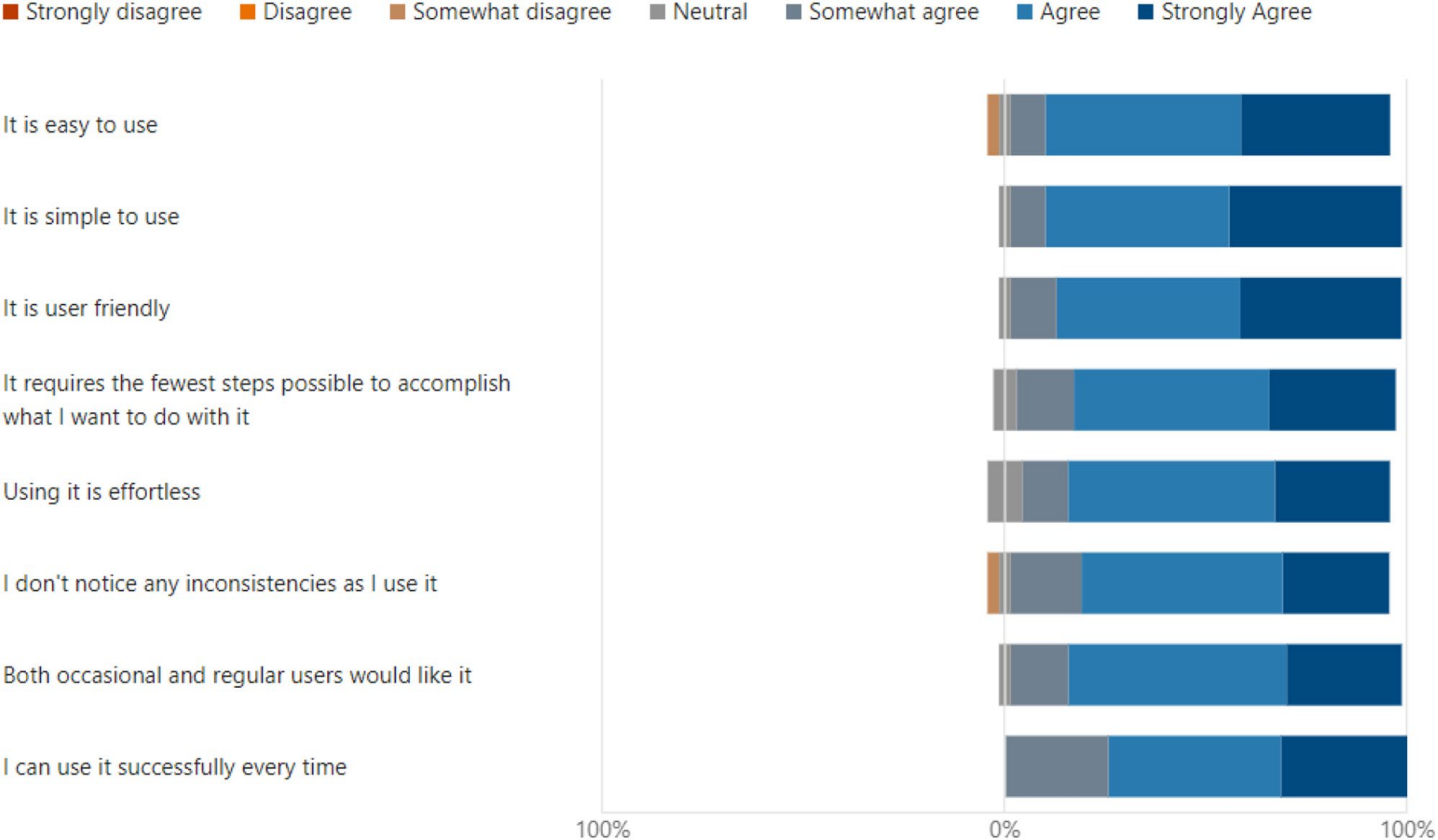
Results for “Ease of Use”

**Fig. 6:**
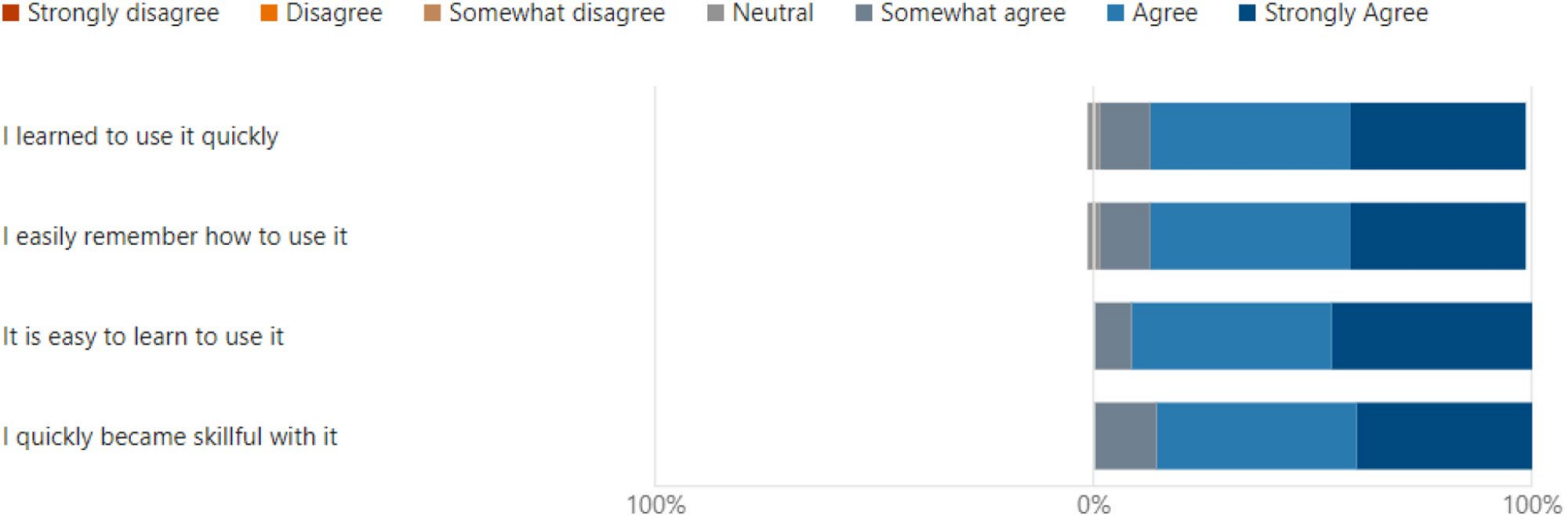
Results for “Ease of Learning”

**Fig. 7:**
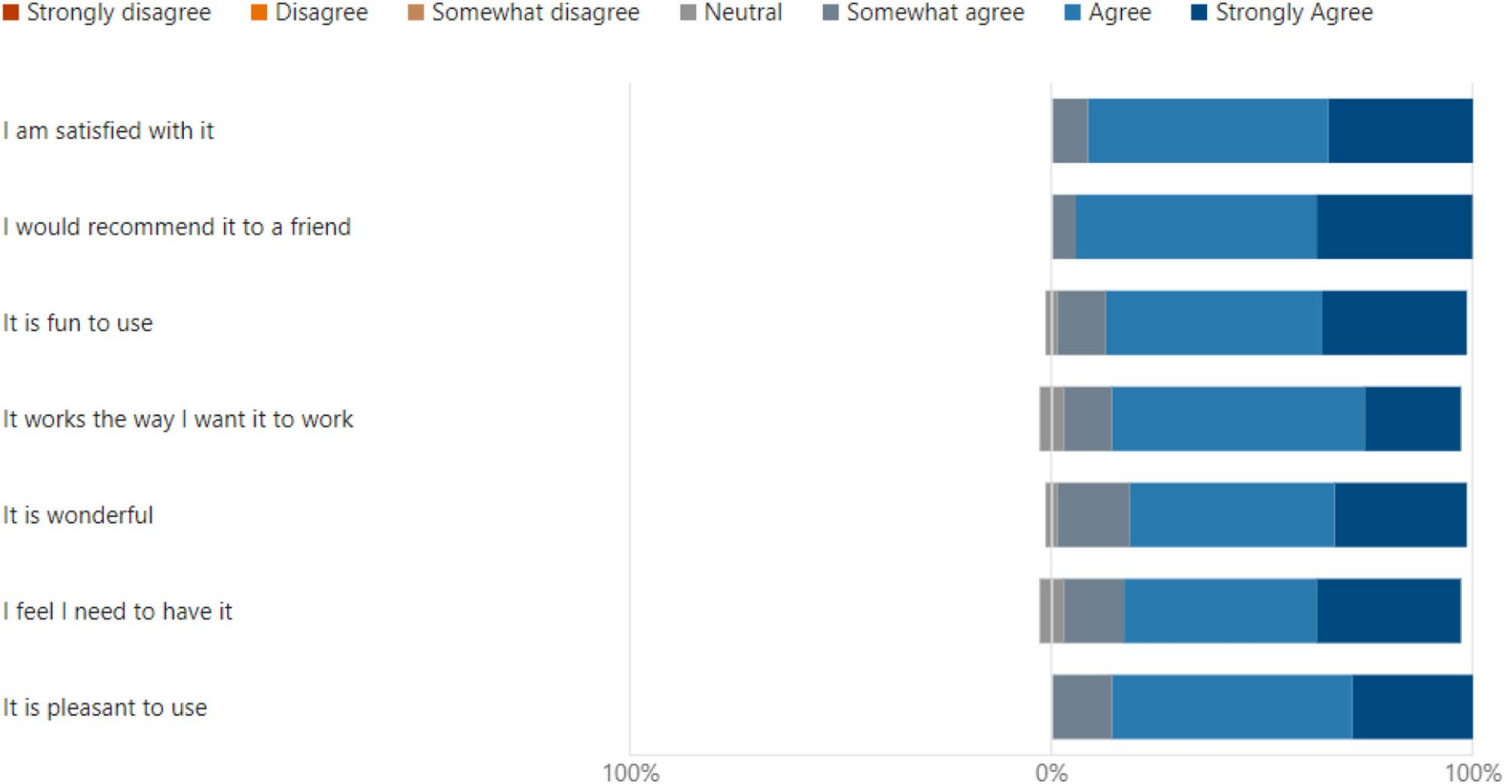
Results for “Satisfaction”

Wilcoxon test was chosen as a more appropriate analysis for the data’s distribution. This statistical approach and adjustments accounted for non-parametric data and multiple comparisons. Data analysis using Wilcoxon signed rank test with Bonferroni correction found significant differences between ‘Uselessness’ and other domains, which demonstrated the perceived usability of VDCs as an educational tool in the clinical setting. Importantly, uselessness was rated significantly higher compared to ‘Ease of use (*p* < 0.001), ‘Ease of learning’ (*p* < 0.001) and satisfaction (*p* < 0.001). (Table 1) No significant differences were observed between ‘Ease of use, ease of learning and satisfaction’ *p* > 0.05. The results for the usefulness domains suggest that although the tool is effective in helping the user achieve their task, there is still room for improvement in making it more user-friendly and easier to learn as well as in overall user satisfaction (Fig. 4).The study found no significant difference between the ease of use, ease of learning and satisfaction domains, which portrays similarly positive user experience across those domains (Figs. 5, 6 and 7). Our analysis has provided an understanding of student perception across the various aspects and domains of the tool, indicating varying perceptions of the tool’s usability in a clinical education context. The significant differences between some domains highlight variability in end-user experience, Overall, the analysis provides insights into student perceptions across various usability domains, highlighting areas for potential refinement and informing future development efforts (Table 1).

**Table 1:** Wilcoxon Signed-Rank test results

Domain Comparison	Wilcoxon Test Statistic	Unadjusted *p*-value	Bonferroni Adjusted *p*-value
Usefulness vs. Ease of Use	32.0	0.000010	0.000062
Usefulness vs. Ease of Learning	8.0	0.000000	0.0000006
Usefulness vs. Satisfaction	0.0	0.000000	0.000000
Ease of Use vs. Ease of Learning	132.0	0.065515	0.393091
Ease of Use vs. Satisfaction	47.0	0.000077	0.000464
Ease of Learning vs. Satisfaction	142.0	0.065515	0.393091

Table 1: Comprehensive comparison of all the domains of the "USE Questionnaire" in the Virtual Dental Clinics

## Discussion

This paper describes the effective implementation of a virtual clinical reasoning and learning environment in a dental clinic. The VDC has shown great validity and potential for practice-based education where effective clinical and psychomotor skills combine with theoretical knowledge to effectively constitute a diagnosis and treatment plan [24]. Incorporating simulation-based clinical scenarios through VDCs into the curriculum can provide students with multimodal learning opportunities over time. Since the virtual dental clinical cases are made available on their eLearning platform, they are an effective tool for self-study and self-evaluation. The students can re-visit these at different points in time, and their understanding of the clinical scenarios can improve over time. A study conducted by Ayaz et al. [25] found that further enhancements in simulation-based training should be investigated. One of the major advantages of developing these Virtual Dental Clinics is that the learners can repeatedly practice and re-assess their learning while working in a safe and risk-free environment and that is what the current study emulated.

**Fig. 8:**
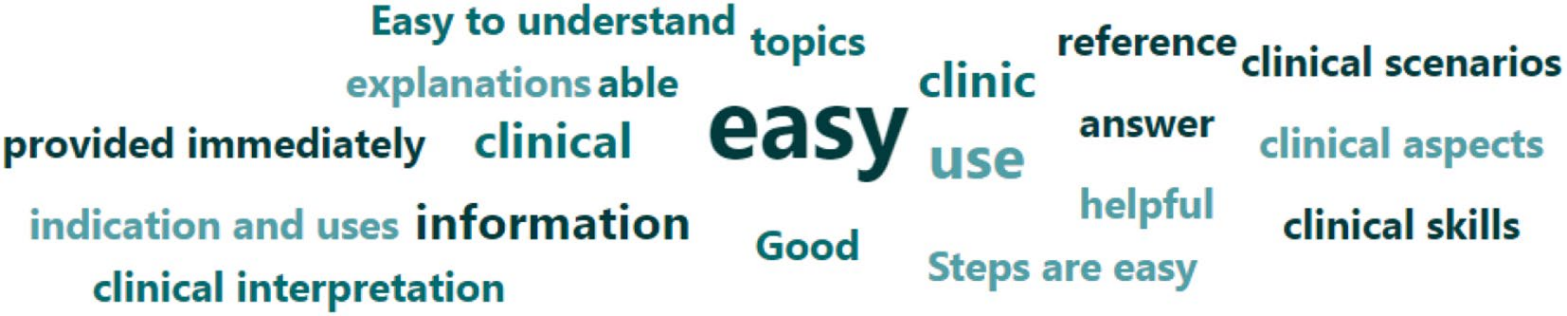
Students suggested positive and negative aspects for the VDCs

The USE questionnaire has previously been a validated and used tool in various studies [20, 21]. This was the first time it was used in a study in dental simulation education. It has been a useful tool as assessed in the current study, it was comprehensive and covers all aspects that needed to be assessed in the present study. The tool also incorporated areas for user feedback. (Fig. 8) One of the core features of adult learning is that the adult learners need to be actively involved in the process of learning according to Bryan et al. [26, 27]. This assists in modifying and re-structing VDCs to incorporate student feedback when more clinical scenarios are developed in this format.

Learning outcomes and objectives to be achieved were also given at the start of each case. This was in line with the Program and course learning outcomes as defined by the Malaysian Qualifying Agency (MQA). This is important so that the objectives for each clinical scenario are aligned to the courses within the domain of each VDC. Our results showed that most students found the virtual dental clinic to be a useful tool, easy to use, and a more user-friendly tool for learning, as reported by Figs. 4, 5 and 6. Many students reported being highly satisfied, while a few were neutral to using the VDC as a learning tool, as reported in Fig. 7. Our results are similar to Wu et al., where the students also found that VDC was a useful tool that was easy to use, provided immediate feedback and a good source of understanding clinical situations and interpretations (Fig. 8) [2]. This underscores the importance of multimodal teaching strategies in dental education, which aim to equip students to work safely, independently, and effectively before graduation [3, 28].

These multimodal strategies can be pre-clinical practice on simulators, self-study, case-based learning, didactic lectures, and problem-based teaching methods. Using various forms of teaching also introduces the students to the more complex clinical cases they may not encounter during their undergraduate training, preparing them for the future. The students improve their diagnostic and clinical skills with the help of pre-clinical and clinical practice, self-reflection and faculty feedback, all of which are essential to improving clinical skills. There are six levels of learning according to the Blooms taxonomy wheel, which are “knowledge, application, comprehension, analysis, synthesis and evaluation [29].

Developing excellent clinical abilities also requires the clinicians’ capacity to objectively self-examine outcomes, identify strengths, weakness and areas for improvement, and implement strategies to accomplish these goals [3]. Identifying fundamental clinical mistakes comes with developing a student’s self-reflection capabilities, allowing for better outcomes.

**Fig. 9:**
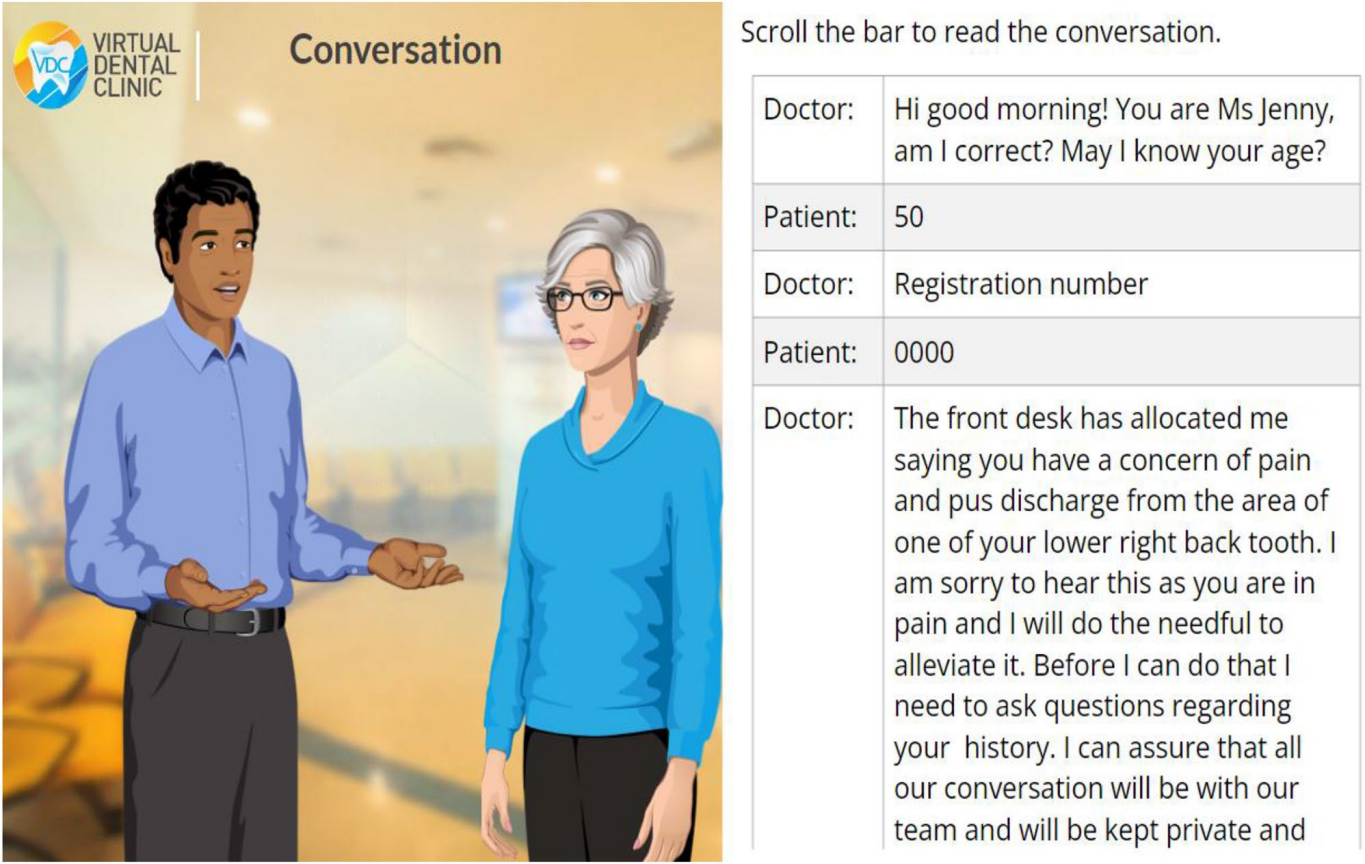
A Sample of the conversation in one of the Virtual Dental Clinic

The VDCs developed in our system incorporated virtual patients with realistic case scenarios. These included conversations between dentists and patients, which trains the students in the art of history taking. The choice of words used and the manner of communicating with patients is an important communicative skill which develops with practice (Fig. 9).

In the current study, the clinical steps to reach a diagnosis were also incorporated with short interactive quizzes, this would make the VDC unique in a way that the user can only proceed with the next step once the quiz was attempted. The quiz also gave the participants an explanation to the correct and incorrect answers. In a study conducted by Gardener et. al [28] it was noted that the senior students found the VDC useful in promoting critical thinking skills and understanding the concepts of diagnosis and treatment planning better. The VDC thus enhanced the teaching done in the early years of dental education.

In the present study, the VDC included various subjects taught in the advanced years of undergraduate dental education. The topics included Oral Medicine, Oral Radiology, Endodontics and Maxillofacial surgery. This contrasts with Wu et al.‘s study, in which the topics covered in VDC were limited to tooth caries and pulpitis [2].

### Limitations and future recommendations

One of the limitations of the present study was that the sample size was much smaller than anticipated. This is suggestive of the students’ time being utilised in more didactic learning, and students would not access the VDCs until closer to their assessment time. Another limitation was that a comparison between student groups was not made. We recommend that future studies should assess the difference in understanding levels of students from different levels of undergraduate dental training to assess the VDCs effectiveness as a helpful tool for more advanced or early-stage cohorts. Including more sub-specialities in dentistry or integrating a few specialities together would make the clinical cases more realistic and that would be more useful to the students when they are preparing for their clinical training. The VDC can also be incorporated before a seminar or case-based learning for the learners, for them to be more prepared for the didactic session, and the cases can be discussed in detail with the subject specialists.

The small sample size (*n* = 29) limits the generalizability of our results to the wider population and might diminish the study’s statistical significance. More research with bigger and more diversified population sample is required to strengthen the research findings and make the study more generalizable.

In essence these Virtual dental clinics can also be used as scenarios to be developed in Virtual Reality (VR) Simulators. With the enhanced use of haptics in VR simulators that are now available for dental education, the VDCs would provide avenues for further improvement in the students’ psychomotor skills in more realistic situations.

The undergraduate students found the virtual dental clinic to be a useful self-learning tool in dentistry in our study. This suggests that VDCs can possibly be used for postgraduate students and should incorporate additional subjects in clinical dentistry, involving more complex and multi-disciplinary clinical scenarios, that students may not experience in their everyday dental practice. This would assist students in preparing for their examinations and can be repeated with self-assessment to improve clinical diagnosis and management.

## Conclusion

This study has provided an understanding of student perception across the various aspects of usefulness, satisfaction, ease of use, and ease of learning in virtual dental clinics. The significant differences between Usefulness domain and others highlight variability in end-user experience, provides a starting point for future improvements and optimization of end-user experience. Overall, the analysis provides insights into student perceptions across various usability domains, highlighting areas for potential refinement and informing future development efforts.

Virtual dental clinics are promising tool for simulation-based teaching and should be incorporated at the pre-clinical and clinical years. This would give the students greater flexibility and exposure as well as increase their confidence before they encounter real-life patients with complex clinical conditions.

## Data Availability

No datasets were generated or analysed during the current study.

## Abbreviations

VDCs: Virtual Dental Clinics
